# Treatment prescribing patterns in a cohort of patients with juvenile idiopathic arthritis (JIA). Data from the childhood arthritis prospective study (CAPS)

**DOI:** 10.1186/1546-0096-12-S1-P23

**Published:** 2014-09-17

**Authors:** Rebecca Davies, Roberto Carrasco, Helen E Foster, Eileen Baildam, Alice Chieng, Joyce E Davidson, Yiannakis Ioannou, Lucy Wedderburn, Wendy Thomson, Kimme Hyrich

**Affiliations:** 1 University of Manchester, Manchester,UK; 2 -, -, UK

## Introduction

Juvenile idiopathic arthritis (JIA) is a heterogenous disease, classified according to the International League of Associations for Rheumatology (ILAR). Initial treatment is based largely on disease severity; intra-articular injections for oligoarthritis, methotrexate (MTX) for polyarthritis and systemic presentations. The recent licensing of biologic therapies for use in JIA has revolutionised treatment of the disease. It is not currently known what proportion of children who present with polyarthritis will require biologic therapy. Although not studied formally, it is recognised a proportion of children with oligoarthritis will also require systemic therapy to control symptoms.

## Objectives

To describe prescribing patterns in JIA over the first 3 years on presentation to rheumatology.

## Methods

Children with at least 3 years of follow-up within the Childhood Arthritis Prospective Study (CAPS), a prospective observational inception study of inflammatory arthritis, were included.

For analysis, children were placed into one of 4 groups based on physician-assigned ILAR category and number of active joints at first presentation (baseline): oligoarthritis, polyarthritis, systemic (sJIA) and enthesitis-related arthritis (ERA). All treatment exposures were categorised into NSAID, intra-articular steroids, disease modifying anti-rheumatic drug (DMARD) including MTX and sulphasalazine (SSZ) and biologics including adalimumab (ADA), etanercept (ETN), infliximab (INF), and tocilizumab (TCZ).

## Results

790 children were included originally (406 oligoarthritis, 221 polyarthritis, 42 sJIA and 43 ERA). Of these, 78 had missing ILAR and were excluded, leaving 712 children. Over a 3 year period, almost 100% of children with polyarticular presentation and 50% with oligoarthritis went on to receive a DMARD. 44% with polyarthritis and 17% with oligoarticular presentation also received a biologic (Table). The most recent ILAR category among children with oligoarticular onset who received a biologic comprised 39% extended, 19% polyarthritis, 4% ERA, 11% other subtypes; 27% had persistent oligoarthritis. All sJIA patients were treated with DMARDs with 36% having biologics . 63% of ERA patients receive a DMARD with 26% going on to receive a biologic. Table 1.

**Table 1:** 

Arthritis pattern at presentation	N	Ever had a DMARD, n(%)	Ever had a biologic, n(%)
**Oligoarthritis**	406	204 (50)	70 (17)

**Polyarthritis**	221	217 (98)	98 (44)

**Systemic arthritis**	42	42 (100)	15 (36)

**Enthesitis-related arthritis**	43	27 (63)	11 (26)

## Conclusion

Over a three year period almost all patients with polyarthritis received treatment with MTX and almost 50% also received a biologic therapy. A high proportion of children presenting with oligoarthritis also went on to receive DMARDs and biologics, many children for persistent oligoarthritis. This is despite the lack of clinical trial evidence for effectiveness in this subtype. Further studies on the efficacy/effectiveness in this subtype should be undertaken to ensure appropriate use of advanced therapies in this population.

## Disclosure of interest

None declared.

